# Adult-Onset Still’s Disease Presenting as Aseptic Meningitis: A Case Report and Literature Review

**DOI:** 10.7759/cureus.79999

**Published:** 2025-03-03

**Authors:** Raja Bakhsh, Khaled Dairi, Mohammad Hamid Alabdali, Kholood Almehmadi, Osama Alhindi, Reem Alhassani, Hanan Alkaabi, Anfal Alsharif

**Affiliations:** 1 Pharmacology and Therapeutics, King Faisal Hospital, Makkah, SAU; 2 Rheumatology, King Faisal Hospital, Makkah, SAU; 3 Hematology, King Faisal Hospital, Makkah, SAU; 4 Internal Medicine, King Faisal Hospital, Makkah, SAU

**Keywords:** adult-onset still's disease, aseptic meningitis, cerebrospinal fluid, macrophage activation syndrome, neurological manifestations, rheumatology, systemic disease

## Abstract

Adult-onset Still’s disease (AOSD) is a rare systemic inflammatory disease that usually presents with nonspecific symptoms, making early diagnosis difficult. It presents with high spiking fevers, salmon-colored rashes, and arthritis. However, atypical presentations of AOSD exist, as seen in this case, where AOSD manifested as aseptic meningitis, further complicating the diagnosis. Given its rarity and symptom overlap with infections and autoimmune diseases, AOSD poses significant diagnostic challenges. This is a case report of a 51-year-old Saudi Arabian female who suffered several weeks of fever and rash and was initially treated for an allergic reaction. However, the persistence of symptoms led to further investigation. A rheumatology consultation suggested AOSD based on persistent fever, leukocytosis, and negative cerebrospinal fluid cultures. Laboratory findings, including a raised ferritin level, further supported the diagnosis. She was effectively treated with corticosteroids, immunoglobulins, and other immunosuppressive drugs, resulting in complete symptom resolution. This case highlights the importance of considering AOSD in patients with aseptic meningitis and persistent systemic inflammation, reinforcing the need for timely diagnosis and intervention.

## Introduction

Adult-onset Still’s disease (AOSD), first reported in 1971, is a rare systemic inflammatory condition characterized by nonspecific symptoms such as fever, rash, and arthritis, leading to frequent diagnostic delays [1,2]. The fever typically follows a quotidian pattern, meaning it occurs daily or at times, peaks twice daily, and typically precedes other clinical features. Joint involvement is characteristically recurrent, with arthritis starting as mild and localized but over time evolving into severe polyarthritis [3]. While neurological involvement in AOSD is uncommon, aseptic meningitis has been reported in a subset of cases, posing unique diagnostic challenges due to its overlap with infectious and autoimmune conditions.

Epidemiologically, AOSD is a rare disease. According to Riaz et al. (2023), the prevalence and incidence of AOSD are reported as 0.16-0.62 annually per 100,000 people and 0.73-6.77 per 100,000 people, respectively [4]. The prevalence of the disease estimated by recent studies is 3.9 per 100,000 in Japan and 6.77 per 100,000 in Turkey, showing a regional variation in the incidence rates. AOSD is not common, and this low frequency contributes to significant diagnostic difficulties because its clinical presentation commonly resembles that of infections or malignancies, thereby leading to easy misdiagnosis and delay in the right treatment. Diagnosis is usually performed by exclusion, and the lack of specific biomarkers makes diagnosis even more difficult, which increases the burden of the disease in general [5].

AOSD manifests systemic inflammation with very high elevated levels of inflammatory markers, leukocytosis, and very high ferritin levels. AOSD exhibits a bimodal distribution, peaking between ages 15 and 25 and then between ages 36 and 46 years, as seen in Ref. [6]. The great rarity of AOSD itself hampers clinical practice by limiting the performance of large studies or clinical trials. Consequently, healthcare providers may face challenges in recognizing and managing this complex condition effectively [7].

Aseptic meningitis refers to the inflammation of the meninges, defined by cerebrospinal fluid (CSF) pleocytosis with a cell count of ≥5 cells/mm³, in the absence of bacterial, viral, fungal, or parasitic infection. The etiology of aseptic meningitis can be categorized into three primary groups: systemic disorders with meningeal involvement, such as sarcoidosis and Behçet's disease; drug-induced aseptic meningitis, triggered by various medications including non-steroidal anti-inflammatory drugs (NSAIDs), antibiotics, intravenous immunoglobulin, and monoclonal antibodies; and neoplastic meningitis, associated with malignancies [8].

Aseptic meningitis is the most common neurological presentation of AOSD and was seen in 64.3% of cases with neurological manifestations. Other less common neurological manifestations of this disease include cranial nerve palsy, encephalitis, and cerebral infarction [9]. Aseptic meningitis of AOSD diagnosis is challenging as it presents similarly to infectious meningitis and other connective tissue diseases such as systemic lupus erythematosus and Behçet's disease. These diagnostic challenges are compounded by the limitations of the diagnostic modalities, effects of drugs, and similarities in clinical features among these conditions. In such instances, when initial differential diagnoses do not yield a clear result, broad-spectrum antibiotics are often empirically started. However, if the treatment fails and CSF examination is ruled out for infectious etiologies, AOSD-related aseptic meningitis should be suspected and glucocorticoid therapy should be started [10].

Aseptic meningitis in AOSD illustrates poorly understood pathogenesis. As the patients are in a hyperinflammatory state, it would be postulated that the activated neutrophils may cross the blood-brain barrier and cause the development of meningitis. CSF study usually demonstrates a mixed response, obscuring the difference from infectious meningitis, commonly characterized by a high neutrophil count associated with low glucose levels [11]. The basis for the diagnosis is the collection of clinical history, detailed physical examination, and serological detection of autoantibodies. Clinical suspicion and complete diagnostic assessment are important because of the systemic complexity of AOSD and the infrequent occurrence of neurological complications for its proper management [12].

AOSD is mainly characterized by nonspecific symptoms and a lack of specific biomarkers. Hence, it presents a huge challenge for diagnosis. Neurological involvement, though rare, constitutes the severe presentation that further complicates the accurate diagnosis and the effective management of the disease. As other diseases have overlapping features clinically, with AOSD being extremely rare, research studies are required to delineate neurological involvement better in order to refine diagnostic strategies. This case describes a 51-year-old female diagnosed with AOSD, where the case presentation is by aseptic meningitis, which highlights the difficulties involved in diagnosis. As such, a high level of suspicion should be appropriately matched with elaborate diagnostic strategies to identify AOSD among its close mimickers. This case presentation aims to elaborate on the clinical course, the method of diagnosis, and the therapeutic response of this highly uncommon neurological manifestation of AOSD to add to the literature.

## Case presentation

A 51-year-old female from Saudi Arabia, with no significant medical history, was admitted to King Faisal Hospital in Makkah City by ambulance with a documented fever of 38°C. She complained of recurrent episodes of fever over the past three weeks, associated with an itchy skin rash that improved after each episode of fever.

Clinical history and presentation

The patient had been treated for three weeks with antihistamines (cetirizine) suspecting an allergic reaction to an unknown allergen. However, symptoms persisted. She also showed significant fatigue, night sweats from time to time, headaches, and joint stiffness lasting less than 30 minutes. There was no family history of autoimmune diseases.

On arrival at the emergency department, a brain CT scan was performed which was normal. Laboratory investigation results are documented in Tables 1-3.

**Table 1:** Laboratory Results (Initial) Hematology

Parameter	Result	Reference Range
White blood cells (×10³/μL)	18.69	4-11
Hemoglobin (g/dL)	9.6	12-15
Mean corpuscular volume (fL)	76.3	83-101
Mean corpuscular hemoglobin (pg)	24.7	27-32
Platelet (×10⁹/L)	213	150-400
Neutrophil count (×10³/μL)	88.9	2-7.5
Lymphocyte (×10³/μL)	6.8	1.5-3.5

**Table 2:** Laboratory Results (Initial) Biochemistry ALT, alanine aminotransferase; AST, aspartate aminotransferase; ALP, alkaline phosphatase; LDH, lactate dehydrogenase; CKI, creatine kinase; ESR, erythrocyte sedimentation rate; CRP, C-reactive protein; PT, prothrombin time; PTT,  partial thromboplastin time; INR, international normalized ratio.

Parameter	Result	Reference Range
Sodium (mmol/L)	129.9	137-145
Potassium (mmol/L)	5.2	3.5-5.1
Creatinine (μmol/L)	45.76	46-92
Random glucose (mmol/L)	10.51	4.1-5.9
ALT (U/L)	49.4	0-35
AST (U/L)	106.08	15-46
Albumin (g/L)	31.3	35-50
ALP (U/L)	124.7	38-126
LDH (U/L)	867.6	120-246
CKI (U/L)	58.07	30-135
Calcium (mmol/L)	1.99	2.1-2.55
Phosphorus (mmol/L)	1.11	0.81-1.45
ESR (mm/h)	120	5-20
CRP (mg/dL)	>45	0-1
PT (s)	14	10-14
PTT (s)	25	23-39
INR	1.0	0.85-1.1

**Table 3:** Laboratory Results (Initial) CSF Analysis CSF, cerebrospinal fluid; VDRL, Venereal Disease Research Laboratory.

Parameter	Result	Reference Range
CSF (WBC) (/μL)	5	0-5
CSF microbiology	Negative	-
CSF (glucose) (mmol/L)	5.7	2.2-3.9
CSF (protein) (mg/L)	500	150-450
CSF culture	Negative	-
VDRL	Negative	-

**Figure 1:**
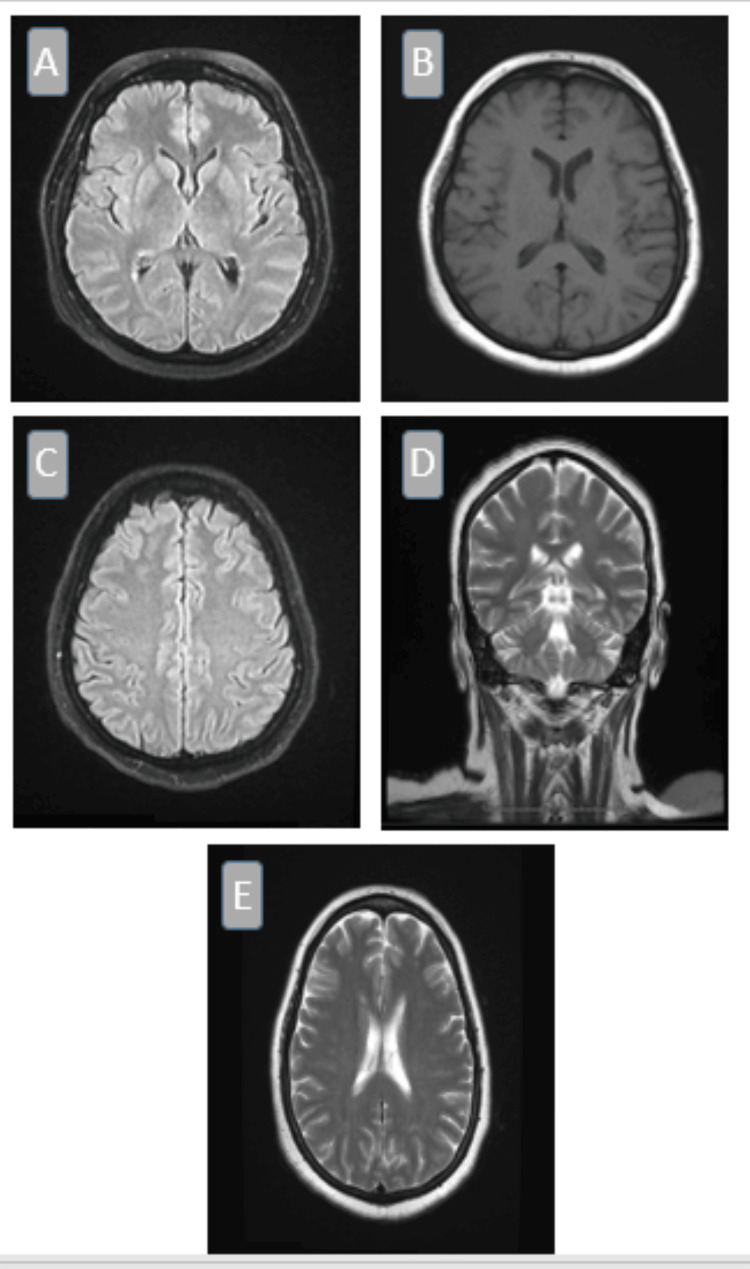
MRI of the brain illustrates abnormal leptomeningeal changes, indicative of potential pathological processes such as inflammation or infection. Images (A) through (E) show various views highlighting abnormal meningeal thickening and patchy enhancement on post-gadolinium FLAIR T2-weighted imaging. (A) Axial FLAIR: Hyperintense leptomeningeal signal abnormalities suggestive of inflammation or infection. (B) Axial T1-weighted (post-contrast): Patchy leptomeningeal enhancement indicating potential meningeal inflammation. (C) Axial FLAIR: Diffuse cortical and subcortical hyperintensities, indicative of meningeal pathology. (D) Coronal T2-weighted: Meningeal thickening along the cortical surface, possibly due to infectious or inflammatory etiology. (E) Axial T2-weighted: Abnormal meningeal thickening with hyperintense areas, supporting the presence of a pathological process. FLAIR, fluid-attenuated inversion recovery.

The initial diagnosis was meningoencephalitis though CSF microbiology was negative. Based on the clinical suspicion of meningitis, the patient was admitted and empiric treatment for meningitis was commenced. Figure 1 highlights the areas of abnormal leptomeninges indicating potential pathological processes such as inflammation or infection. MRI of the brain revealed abnormal meningeal thickening with intravenous contrast and no other significant abnormalities were observed.

Hospital course

Despite ceftriaxone therapy, the patient had a persistent fever and remained leukocytic for 10 days. The infectious disease consultant recommended changing her antibiotic medication to meropenem 2 grams intravenous administration every 8 hours. Nonetheless, she failed to show any clinical improvement and had a rash with spikes of fever. A rheumatology consult suggested the possibility of AOSD presenting as aseptic meningitis. Further laboratory tests, including ferritin level, serum erythrocyte sedimentation rate (ESR), and immunoglobulin levels, including IgG, IgA, and IgM, were ordered. Follow-up laboratory results on April 23, 2024, revealed a significantly elevated ESR (118 mm/h) and ferritin level (35,700 µg/L), along with normal immunoglobulin levels. Complement levels (C3 and C4) were within normal ranges, and autoimmune markers, including antinuclear antibody (ANA) and extractable nuclear antigen (ENA) profile, were negative. Infectious serologies for cytomegalovirus (CMV), Epstein-Barr virus (EBV), parvovirus B19, and hepatitis were also negative. Additionally, the peripheral blood film demonstrated microcytosis, hypochromia, anisocytosis, reactive neutrophil leukocytosis, and thrombocytopenia, with no abnormal cells observed (Table 4).

**Table 4:** Laboratory Results (Follow-Up) ESR, erythrocyte sedimentation rate; CMV, cytomegalovirus; EBV, Epstein-Barr virus; ENA, extractable nuclear antigen; ANCA, anti-neutrophil cytoplasmic antibody; C-ANCA, cytoplasmic ANCA; P-ANCA, perinuclear ANCA.

Parameter	Result	Reference Range
C3 complement (mg/dL)	159.55	88-165
C4 complement (mg/dL)	30.5	14-44
Rheumatoid factor (IU/mL)	10	0-12
ESR (mm/h)	118	5-20
Ferritin (µg/L)	35,700	11-264
IgG (mg/dL)	1,415.54	700-1600
IgA (mg/dL)	218.1	70-400
IgM (mg/dL)	83.87	40-230
Infectious serologies (CMV, EBV, parvovirus B19, hepatitis B and C)	Negative	-
Antinuclear antibody	Negative	-
ENA profile	Negative	-
C-ANCA	Negative	-
P-ANCA	Negative	-
Salmonella	<1:80	0
Brucella	<1:80	0

Additional investigations were performed for disease follow-up and organ function monitoring. The results are summarized in Table 5.

**Table 5:** Laboratory Results (Organ Function Monitoring) BUN, blood urea nitrogen; ALT, alanine aminotransferase; AST, aspartate aminotransferase; ALP, alkaline phosphatase; LDH, lactate dehydrogenase; CKI, creatine kinase; CK-MB, creatine kinase-myocardial band.

Lab	Result	Normal Range
White blood cells (×10³/μL)	8.06	4-11
Hemoglobin (g/dL)	8.1	12-15
Mean corpuscular volume (fL)	75	83-101
Mean corpuscular hemoglobin (pg)	25.3	27-32
Platelet (×10⁹/L)	133	150-400
Sodium (mmol/L)	127	137-145
Potassium (mmol/L)	4	3.5-5.1
BUN (mmol/L)	4.5	2.5-6.1
Creatinine (μmol/L)	62.3	46-92
Calcium (mmol/L)	1.8	2.1-2.55
Phosphorus (mmol/L)	1.01	0.81-1.45
ALT (U/L)	53.6	0-35
AST (U/L)	135.9	15-46
Albumin (g/L)	21.9	35-50
ALP (U/L)	140.6	38-126
LDH (U/L)	1015.1	120-246
CKI (U/L)	143.1	30-135
CK-MB (U/L)	8.5	7-25

The peripheral blood film revealed microcytosis, hypochromia, anisocytosis, reactive neutrophilic leukocytosis, and thrombocytopenia without abnormal cells.

Diagnosis

The diagnosis of macrophage activation syndrome (MAS) secondary to AOSD was confirmed by bone marrow biopsy showing macrophages with hemophagocytic activity.

Management and treatment plan

She was treated with intravenous pulse corticosteroid therapy, IVIG, tocilizumab, methotrexate, and folic acid. The patient remained in complete resolution of symptoms including fever, altered sensorium, and joint pain over the next month. Laboratory markers normalized including a dramatic decrease in the serum ferritin levels (Table 6).

**Table 6:** Key Treatments Administered MAS, macrophage activation syndrome.

Treatment	Dosage and Duration	Notes
Corticosteroids (IV)	Pulse therapy	For initial rapid symptom control
Intravenous immunoglobulin	Standard dose	Adjunct therapy for MAS
Tocilizumab	8 mg/kg every 4 weeks	IL-6 inhibitor for inflammation control
Methotrexate	15 mg weekly	Long-term immunosuppressant
Folic acid	1 mg daily	To counteract methotrexate side effects

Outcomes and follow-up

The patient recovered entirely without any recurrence of the symptoms or laboratory abnormalities during the follow-up visits. It is the case that emphasizes and points out the need to look for rare presentations of AOSD, such as aseptic meningitis, and the prompt diagnosis combined with multidisciplinary management preventing serious complications such as MAS.

## Discussion

This is a rare case presentation of AOSD in a 51-year-old female who presented to the hospital after a typical history of fever, rash, and systemic symptoms. The neurological feature, when it occurs in AOSD, usually presents in the form of aseptic meningitis. Only 10 cases have so far been documented in the literature, with most of them being young with neutrophilic leukocytosis as the CSF finding.

Our patient had an unusual case presentation marked by her age and CSF. All the first-line diagnostic tests including CT scans and analyses of CSF were indeterminate, whereas appropriate empirical treatment for meningitis due to persistent fever and leukocytosis was unwarranted. The diagnosis was confirmed with greatly increased serum ferritin (35,700 µg/L), elevated and persistent inflammatory markers, and hemophagocytosis on bone marrow biopsy. Multimodal therapeutic intervention comprising corticosteroids, intravenous immunoglobulin, tocilizumab, and methotrexate resulted in the complete resolution of symptoms of fever, altered sensorium, and joint pain, along with the normalization of all the laboratory parameters such as serum ferritin levels. The clinical presentation and laboratory results presented above, along with a remarkable therapeutic response to immunosuppressive therapy corroborate the diagnosis of AOSD [13]. According to the Yamaguchi criteria, our case meets the diagnostic criteria for AOSD, which includes major criteria such as spiking fever, arthralgia, typical rash, and leukocytosis, along with minor criteria such as elevated liver enzymes and sore throat. This is further supported by the exclusion of infectious and autoimmune etiologies, confirming AOSD as the final diagnosis. This case aligns with the findings by Zhao et al. (2021), where aseptic meningitis was reported in 64.3% of neurological AOSD cases [11]. However, our patient’s advanced age and initially indeterminate diagnostic tests make this presentation unique. The need for high clinical suspicion and reliance on inflammatory markers like ferritin is crucial, as also emphasized by Eleftheriotis and Skopelitis (2021) [14]. They highlighted the role of persistently elevated ferritin and inflammatory markers in distinguishing AOSD from infectious or autoimmune conditions, a finding that strongly aligns with our observations. Furthermore, our patient’s complete resolution with corticosteroids and tocilizumab reinforces the importance of timely immunosuppressive therapy in AOSD with neurological involvement. The pathophysiology of AOSD-related aseptic meningitis remains poorly understood. However, emerging evidence suggests that excessive immune activation, characterized by overproduction of pro-inflammatory cytokines such as IL-1, IL-6, and TNF-α, plays a pivotal role. The hyperinflammatory state in AOSD may facilitate the migration of activated neutrophils and monocytes across the blood-brain barrier, leading to meningeal inflammation. Additionally, endothelial dysfunction caused by systemic inflammation may contribute to increased vascular permeability, further exacerbating neurological involvement. CSF findings, including neutrophilic pleocytosis and elevated protein levels, support this immune-mediated mechanism rather than direct infectious involvement. Moreover, studies indicate that aseptic meningitis in AOSD may be an early marker of developing MAS, a severe complication characterized by uncontrolled immune activation. The recognition of this interplay is critical for prompt immunosuppressive therapy initiation. Given the diagnostic challenges, a high index of suspicion and timely intervention with corticosteroids or biologics targeting IL-6 (e.g., tocilizumab) are essential in preventing disease progression.

The case report by Ito et al. (2019) adds to the literature on the disease of AOSD, with particular attention given to its neurological manifestations. However, it contrasts with our case, as Ito et al. describe an elderly patient presenting primarily with neurological symptoms, whereas our patient exhibited systemic features of AOSD in addition to aseptic meningitis. This comparison highlights the variability in AOSD presentations, reinforcing the importance of a comprehensive diagnostic approach [15].

The study by Risal et al. (2023) described the case of a 78-year-old female initially diagnosed with tuberculous meningitis. However, based on her poor response to therapy and persistently high levels of inflammatory markers, a further diagnosis of AOSD was established. Subsequently, she showed remarkable improvement in immunosuppressive treatment. Like our case, the diagnostic challenge in differentiating AOSD from infectious etiologies was a key issue, emphasizing the need for a thorough evaluation of persistent inflammation and clinical course. The overlap with infectious syndromes remains a significant concern, and our case further supports the importance of considering AOSD in the differential diagnosis of prolonged febrile illnesses with neurological involvement [6].

Several cases highlight the presentation of AOSD with aseptic meningitis, often leading to delayed diagnosis. For instance, a young female presenting with meningeal signs and CSF abnormalities was later diagnosed with AOSD after ruling out infections and observing a positive response to NSAIDs [16]. However, our case is distinct due to the patient’s older age and prolonged indeterminate diagnostic phase, reinforcing the need for alternative diagnostic pathways in atypical AOSD presentations. Corticosteroids remain the mainstay of treatment for AOSD with neurological involvement. Advanced cases unresponsive to steroids have shown improvement with biological agents like tocilizumab, targeting IL-6 [17] (Table 7).

**Table 7:** Application of Yamaguchi Criteria and Comparison With Similar Cases RF, rheumatoid factor; ANA, antinuclear antibody; AST, aspartate aminotransferase; ALT, alanine aminotransferase; AOSD, adult-onset Still’s disease.

Yamaguchi Criteria	Current Case (51-Year-Old Female)	Zhao et al. (2021) [11] (Neurological AOSD Cases)	Eleftheriotis and Skopelitis (2021) [14]	Ito et al. (2019) [15] (Elderly AOSD)
Major criteria				
Spiking fever ≥39°C for ≥1 week	Present	Present	Present	Present
Arthralgia for ≥2 weeks	Present	Present	Present	Absent
Characteristic salmon-pink rash	Present	Present	Absent	Absent
Leukocytosis (≥10,000/mm³, mainly neutrophils)	Present (18.69 × 10³/μL)	Present	Present	Present
Minor criteria				
Sore throat	Absent	Present	Absent	Absent
Lymphadenopathy/splenomegaly	Absent	Present	Present	Present
Abnormal liver function tests	Mildly elevated AST/ALT	Present	Present	Present
Negative RF and ANA	Negative RF and ANA	Negative RF and ANA	Negative RF and ANA	Negative RF and ANA
Exclusion criteria				
Infection	Ruled out	Ruled out	Ruled out	Ruled out
Malignancy	Ruled out	Ruled out	Ruled out	Ruled out
Other rheumatic diseases	Ruled out	Ruled out	Ruled out	Ruled out
Unique aspects	Advanced age, initially indeterminate diagnostic tests, delayed recognition of AOSD	64.3% had aseptic meningitis	Elevated ferritin and inflammatory markers were key for diagnosis	Presented with only neurological symptoms, lacked systemic AOSD signs

## Conclusions

This case report highlights the diagnostic challenge of AOSD presenting as aseptic meningitis, emphasizing the importance of a high index of clinical suspicion in diagnosing rare manifestations. Initial treatment targeted the infectious causes, but the persistence of symptoms and elevated inflammatory markers, including marked hyperferritinemia, led to the diagnosis of AOSD. Accurate diagnosis requires a comprehensive approach and multidisciplinary interaction to distinguish AOSD from other conditions. The findings of this case highlight the need for increased awareness among clinicians, as recognizing atypical presentations of AOSD can aid in timely diagnosis and appropriate management, ultimately improving patient outcomes. The timely implementation of glucocorticoid therapy facilitated clinical improvement, underscoring the importance of early detection and intervention in complex cases.

## References

[1] Macovei LA, Burlui A, Bratoiu I (2022). Adult-onset Still's disease-A complex disease, a challenging treatment. Int J Mol Sci.

[2] Castañeda S, Blanco R, González-Gay MA (2016). Adult-onset Still's disease: Advances in the treatment. Best Pract Res Clin Rheumatol.

[3] Jamilloux Y, Gerfaud-Valentin M, Martinon F, Belot A, Henry T, Sève P (2015). Pathogenesis of adult-onset Still's disease: New insights from the juvenile counterpart. Immunol Res.

[4] Riaz M, Arshad A, Baqir SM, Isra A (2023). Clinical spectrum of adult-onset Still’s disease in patients presenting at a tertiary care hospital in Pakistan: A 16-year retrospective descriptive study. Research Square.

[5] Correll CK, Binstadt BA (2014). Advances in the pathogenesis and treatment of systemic juvenile idiopathic arthritis. Pediatr Res.

[6] Risal U, Dhungana K, Ghimire M (2023). Adult-onset Still's disease in an elderly patient presenting as aseptic meningitis: A case report. Clin Med Insights Arthritis Musculoskelet Disord.

[7] Efthimiou P, Kontzias A, Hur P, Rodha K, Ramakrishna GS, Nakasato P (2021). Adult-onset Still's disease in focus: Clinical manifestations, diagnosis, treatment, and unmet needs in the era of targeted therapies. Semin Arthritis Rheum.

[8] Shukla B, Aguilera EA, Salazar L, Wootton SH, Kaewpoowat Q, Hasbun R (2017). Aseptic meningitis in adults and children: Diagnostic and management challenges. J Clin Virol.

[9] Gopalarathinam R, Orlowsky E, Kesavalu R, Yelaminchili S (2016). Adult onset Still's disease: A review on diagnostic workup and treatment options. Case Rep Rheumatol.

[10] Gerfaud-Valentin M, Jamilloux Y, Iwaz J, Sève P (2014). Adult-onset Still's disease. Autoimmun Rev.

[11] Zhao M, Wu D, Shen M (2021). Adult-onset Still's disease with neurological involvement: A single-centre report. Rheumatology (Oxford).

[12] Kadavath S, Efthimiou P (2015). Adult-onset Still's disease-Pathogenesis, clinical manifestations, and new treatment options. Ann Med.

[13] Adachi S, Takase-Minegishi K, Maeda A (2023). Risk of macrophage activation syndrome in patients with adult-onset Still's disease treated with IL-1 and IL-6 inhibitors: A meta-analysis and single-center experience. Rheumatol Ther.

[14] Eleftheriotis G, Skopelitis E (2021). Case Report: Intracranial hypertension in an adult-onset Still’s disease patient initially presented with prolonged fever. F1000Research.

[15] Ito N, Takahashi M, Miwa Y, Kagami S, Hayakawa H, Inaba A, Orimo S (2019). Adult-onset Still's disease presenting with aseptic meningitis as the first symptom in an elderly patient. eNeurologicalSci.

[16] Sisselman SG (1999). Adult onset Still's disease presenting as aseptic meningitis in a young healthy female. Del Med J.

[17] Sabnis GR, Gokhale YA, Kulkarni UP (2011). Tocilizumab in refractory adult-onset Still's disease with aseptic meningitis--Efficacy of interleukin-6 blockade and review of the literature. Semin Arthritis Rheum.

